# Portable all-fiber dual-output widely tunable light source for coherent Raman imaging

**DOI:** 10.1364/BOE.10.004437

**Published:** 2019-08-05

**Authors:** Maximilian Brinkmann, Alexander Fast, Tim Hellwig, Isaac Pence, Conor L. Evans, Carsten Fallnich

**Affiliations:** 1 Institute of Applied Physics, Corrensstr. 2, 48149 Münster, Germany; 2 Refined Laser Systems UG (haftungsbeschränkt), Münster, Germany; 3 Wellman Center for Photomedicine, Massachusetts General Hospital, Boston, MA 02114, USA; 4 Cells-in-Motion Cluster of Excellence (EXC 1003 - CiM), University of Münster, Münster, Germany; 5 Shared first author

## Abstract

We present a rapidly tunable dual-output all-fiber light source for coherent Raman imaging, based on a dispersively matched mode-locked laser pumping a parametric oscillator. Output pump and Stokes pulses with a maximal power of 170 and 400 mW, respectively, and equal durations of 7 ps could be generated. The tuning mechanism required no mechanical delay line, enabling all-electronic arbitrary wavelength switching across more than 2700 ${\text{cm}}^{-1}$ in less than 5 ms. The compact setup showed a reliable operation despite mechanical shocks of more than 25 $\text{m}/{\text{s}}^{2}$ and is, thus, well suited for operation in a mobile cart. Imaging mouse and human skin tissue with both the portable light source and a commercial laboratory-bound reference system yielded qualitatively equal results and verified the portable light source being well suited for coherent Raman microscopy.

## 1. Introduction

Coherent Raman scattering (CRS) is in particular advantageous for *in vivo* investigations or diagnostic imaging, due to the non-destructive, label-free and chemically selective nature of CRS and the accordingly low amount of required sample preprocessing. Despite its use in cancer diagnostics [1,2], stain-free histopathology [3], pharmaceutical research [4] and other medically-relevant fields, diagnostic CRS systems are rarely found in clinical settings, mainly due to the complexity of the required light source. An ideal light source for CRS provides two synchronized pulse trains, whose difference in optical wavenumber is tunable between 500 and 3550 ${\text{cm}}^{-1}$ in order to enable probing of different vibrational resonances. These pulse trains can be in the femtosecond or picosecond regime, with picosecond pulses preferred as their low spectral bandwidth provides high spectral selectivity for both coherent anti-Stokes Raman scattering (CARS) as well as stimulated Raman scattering (SRS), and a lower non-resonant background in CARS imaging [5]. Synchronized pulse trains matching the above requirements can be generated by synchronously pumped optical parametric oscillators (OPO) [6,7], making them an appropriate choice for laboratory applications. While free-space OPO systems have been used in a few clinical settings for studies on fresh human tissue in a stationary manner [8–12], an intraoperative or bed-side application is not compatible with their complexity, footprint and need for a floating table. This has led to many research activities being devoted to instrumentation for easing the clinical adaption of CRS, including endoscopes [13] and microscopes [14]. A mobile and automated CARS tomograph for imaging in clinical environments was presented by König et al. [15] where the required dual-color laser pulses were derived from a titanium-sapphire laser pumping a photonic-crystal fiber for supercontinuum generation. However, supercontinuum generation suffers from a low spectral power density (< 20 mW/nm) and the need of a tunable, narrowband spectral filter to extract pulses at different wavelengths. Tunable light sources based on supercontinuum therefore typically offer less than 5 mW of output power [16], whereas up to 50 mW are typically used for *in vivo* imaging. Furthermore, the free-space fiber coupling and beam delivery required active beam stabilization in order to enable the operation offside a floating table.

In contrast, all-fiber light sources are mechanically stable and require almost zero effort for alignment or stabilization. Leveraging the advantages of fiber optics, an integrated clinical system for CRS was developed to be housed in a portable cart for intraoperative optical histology by Orringer et al. [12]. However, as the system made use of synchronized ytterbium-doped and erbium-doped fiber lasers, only vibrational resonances in the CH-stretch region (2700 to 3100 ${\text{cm}}^{-1}$) were accessible due the limited gain bandwidth of the fiber lasers [17,18]. For the same reason, a recently presented fiber-endoscopic CARS spectroscopy system for non-laboratory applications, based on a synchronized ytterbium-doped fiber laser and a diode laser, was limited to the CH-stretch region as well [19]. Although vibrational components in the CH-stretch region in many cases give sufficient contrast for tomographic assessment, many applications focus on vibrational components outside the CH-stretch region. Related applications include screening of deuterated drugs around 2200 ${\text{cm}}^{-1}$ [20], tumor classification in the fingerprint region [21] or visualization of metabolism via deuterium oxide probing [22]. Therefore, all these applications require a widely tunable light source.

Fiber-based OPOs (FOPO) are much more stable than free-space OPOs and exploit the process of four-wave mixing (FWM) for wide wavelength tunability [23–26]. The phase matching condition required for FWM and the accompanied conversion of pump pulses into spectrally up-shifted signal and down-shifted idler pulses is satisfied by an interplay between the fiber’s dispersion and nonlinearity [27]. Typically, a steep phase matching curve is thereby exploited, such that a narrow tuning of the pump wavelength is translated into a wide wavelength shift of the output pulses. In order to maintain the synchronous pumping necessary for operation throughout different changes of repetition rates that occur during wavelength tuning, the timing between the pump laser and FOPO has to be compensated, which is typically achieved by means of a mechanical delay stage in one of the two resonators [23]. Such a free-space delay line compromises the stability of fiber-based systems by being prone to external perturbations and mechanical abrasion. Furthermore, a mechanical delay stage limits the achievable tuning speed in the order of seconds, which is longer than the typical acquisition time in CRS and is considered a bottleneck for multicolor imaging. This problem could be solved in a completely fiber-integrated system by using a fiber-coupled gain-switched laser diode as the pump laser and by adjusting the pump laser’s repetition rate only by electronic means [28]. However, in this work, the FWM gain was spectrally fixed such that the FOPO was tunable only across the fingerprint region due to the fixed emission wavelength of the laser diode close to the zero-dispersion wavelength of the PCF.

The light source presented in this contribution is based on a mode-locked oscillator, which was tunable in wavelength across 40 nm. This mode-locked oscillator synchronously pumped a FOPO with a PCF for FWM which showed a steep phase matching curve such that the gain bandwidth could be shifted across 2700 ${\text{cm}}^{-1}$, spanning the fingerprint, silent and high wavenumber regions by tuning the pump wavelength only. But with a steep phase matching, a large mismatch of repetition rates between the pump laser and the FOPO across the tuning range has to be taken into account.

In order to keep the pump laser with the OPO in synchrony across the entire tuning range without the above mentioned mechanical delay line, a patented delay-free tuning mechanism was introduced which accomplished dispersive matching of both resonators via a chirped fiber Bragg grating (CFBG). This CFBG compensated for the different dispersions of the pump laser and the FOPO resonators such that the repetition frequency change of the pump laser occurring during wavelength tuning related to the dispersion of the cavity was equal to the corresponding change of repetition frequency of the FOPO. As only an electronically tunable fiber-coupled wavelength filter in the pump laser was required for wavelength tuning, the OPO could be completely composed of all-spliced fiber components, yielding a compact and rugged system. In the following, we will present results of the light source’s performance even under mechanical impact and of the validation of its biomedical imaging capabilities in comparison to a commercial state-of-the art laboratory-bound reference light source.

## 2. Portable and widely tunable fiber-based optical parametric oscillator

As shown in Fig. 1

**Fig. 1.**
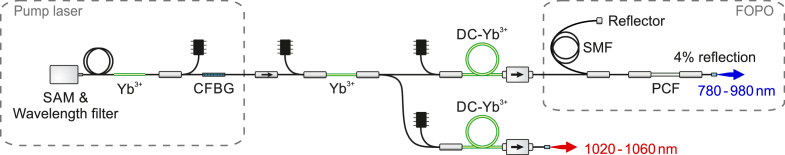
Schematic experimental setup. SAM: saturable absorber mirror; Yb^3+^: Yb-doped fiber; CFBG: chirped fiber Bragg grating; DC-Yb^3+^: double-clad Yb-doped fiber; SMF: single-mode fiber; PCF: photonic-crystal fiber.

, the all-fiber system was based on an ytterbium-doped fiber oscillator, which was mode-locked by a saturable absorber mirror and emitted pulses with a duration of 7 ps and an output power of about 2 mW. The central wavelength of the pulses could be tuned all-electronically between 1020 and 1060 nm in less than one millisecond by a custom-made fiber-coupled electronically tunable filter. The repetition rate of the laser system was 40.5 MHz at a central wavelength of 1040 nm. A specifically chosen CFBG acted as the outcoupler of the pump oscillator (transmission of 25 %) as well as matched the dispersion of the oscillator to the dispersion of the subsequent FOPO. After pre-amplification, the pump pulses seeded two power-amplifiers based on double-clad ytterbium-doped fibers to generate sufficient pulse energy to pump the singly resonant FOPO in one arm and to output the amplified pump pulses from a second arm for the application as Stokes pulses in CRS. The output pump pulses exhibited a power of about 400 mW, a duration of 7 ps (Fig. 2 (a)

**Fig. 2.**
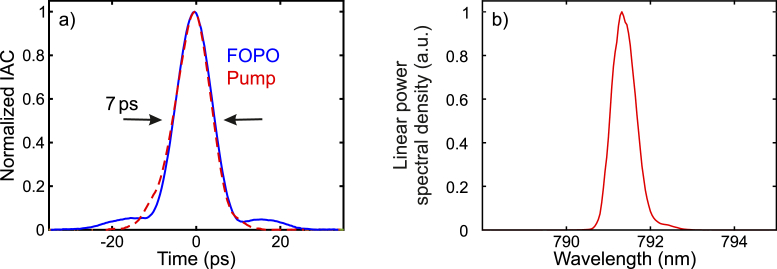
(a) Measured intensity autocorrelation (IAC) traces of the pump and signal pulses. The shown FWHM of 7 ps stayed constant across the tuning range. (b) Exemplary measured spectrum of a signal pulse at 791.5 nm.

), and a spectral width of about 0.6 nm.

The linear cavity of the FOPO was formed by 50 cm of photonic-crystal fiber (NKT Photonics, LMA-PM-5) and about 155 m of single-mode fiber (Nufern, PM780-HP) between a fiber optical retroreflector and a polished FC/PC connector with a reflectivity of about 4 %. According to the resulting optical resonator length, the native repetition rate of the FOPO was 657 kHz, yet, the repetition rate of the output signal pulses was solely determined by and equal to the repetition rate of the pump pulses of 40.5 MHz: At each point of time, multiple signal pulses were circulating in the OPO cavity with a temporal spacing equal to the temporal spacing of the pump pulses. Thus, in analogy to the effect of harmonic mode-locking [29], the OPO was harmonically pumped at 40.5 MHz. The particularly long fiber resonator was chosen in order to form a spectrally narrow dispersive filter in the resonator [28,30], meaning that a fed back signal pulse was temporarily stretched due to the fiber dispersion, such that only a narrow spectral part of the circulating signal pulse overlapped with the next pump pulse and was amplified. Due to this dispersive filtering, the full width at half-maximum (FWHM) bandwidth of the signal pulses was narrowed down to about 12 ${\text{cm}}^{-1}$ (Fig. 2 (b)), which is well suited for highly selective CRS, matching the typical vibrational linewidth in liquid environment. For comparison, shortening the fiber to a length of 5 m, corresponding to a native repetition rate of 40 MHz, in the otherwise unchanged FOPO resulted in a broader dispersive filter and a spectral bandwidth of 31 ${\text{cm}}^{-1}$ without other significant changes of the output parameters.

**Fig. 3.**
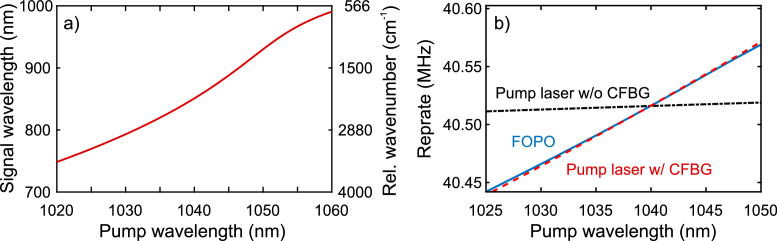
(a) Spectral location of the maximum FWM gain for the signal pulses as a function of the pump wavelength. Left vertical axis shows the signal wavelength, right vertical axis shows the relative wavenumber between the signal and the pump pulses. (b) Repetition rates of the FOPO (blue line) and of the pump laser both with (black dash-dotted line) and without CFBG (red dashed line.

The phase matching curve of the PCF is shown in Fig. 3 (a)

, illustrating that by tuning the pump wavelength from 1020 to 1060 nm the FWM gain for the resonant signal pulses could be shifted from 750 to 980 nm, resulting in a tunable energy difference of pump and signal pulses between 570 and 3300 ${\text{cm}}^{-1}$. As shown in Fig. 3 (b), in this coupled tuning process of pump and signal pulses, the repetition rate of the FOPO changed by 127 kHz, whereas the repetition rate of the pump oscillator without the CFBG changed by 7 kHz only. When we compensated for this mismatch with a free-space delay line in order to maintain synchronous pumping during wavelength tuning, a change of up to 2 cm of the optical path length of either the pump laser or the FOPO was necessary. As mentioned before, such a delay line and the associated free-space fiber coupling were sensitive to misalignment by external perturbations. Therefore, we utilized a specifically chirped fiber Bragg grating to match the repetition frequency change of the pump pulses to the associated repetition frequency change of the resonant signal pulses in the FOPO, as shown by the dashed red line in Fig. 3 (b). In this way, pump and signal pulses stayed inherently synchronized across the complete tuning range and no mechanical alteration of the FOPO or pump resonator was required for tuning the signal wavelength. As an additional benefit, the tuning speed was no longer limited by mechanical inertia, but by a settling time of about 5 ms, which the FOPO required to stabilize at a new wavelength. Consequently, an unprecedented tuning speed of 5 ms per wavelength step was reached, independent of the width of the step up to 2700 ${\text{cm}}^{-1}$.

**Fig. 4.**
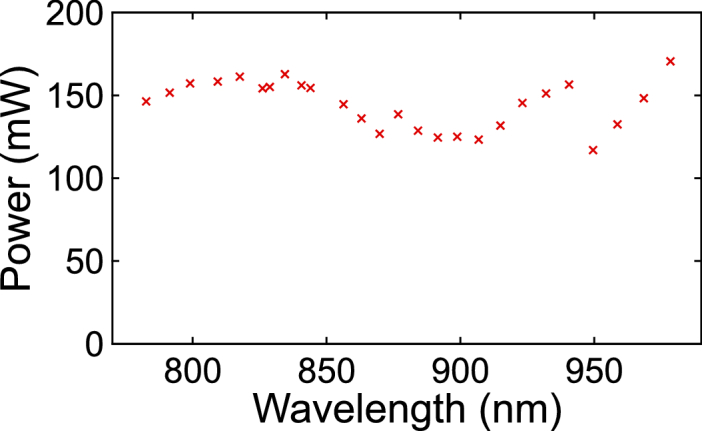
Measured signal power across the tuning range.

The minimum signal output power across the tuning range amounted to 130 mW as shown in Fig. 4

. The FWHM duration of the output signal pulses was measured to be 7 ps across the whole tuning range. A sample autocorrelation trace can be seen in Fig. 2 (a), revealing minor shoulders, which were a result of third order dispersion and the onset of nonlinear effects in the fiber resonator. Despite this minor temporal distortion, 92 % of the signal pulses’ energy overlapped with those of the pump pulses, meaning that signal and pump pulses were well matched in terms of temporal overlap. Although in CRS imaging pulses with a shorter duration of about 3 ps are typically used for a strong signal generation [31], pulses with a duration of 7 ps have been shown to enable *in vivo* CRS with a frame rate of up to 15 Hz [6]. Therefore, we chose the duration of 7 ps as it represents a convenient trade-off between strong signal generation and low nonlinear distortions in our light source as well as in potentially added transport fibers or endoscopes. We have verified by numerical modeling based on the generalized nonlinear Schrödinger equation that the output pulses of our light source with an average power of up to 50 mW can be transported along 2 m of a commercial single-mode large-mode area fiber without a significant reduction of the achievable spectral resolution. For a higher power Kagome lattice hollow core fibers are recommended [13].

**Fig. 5.**
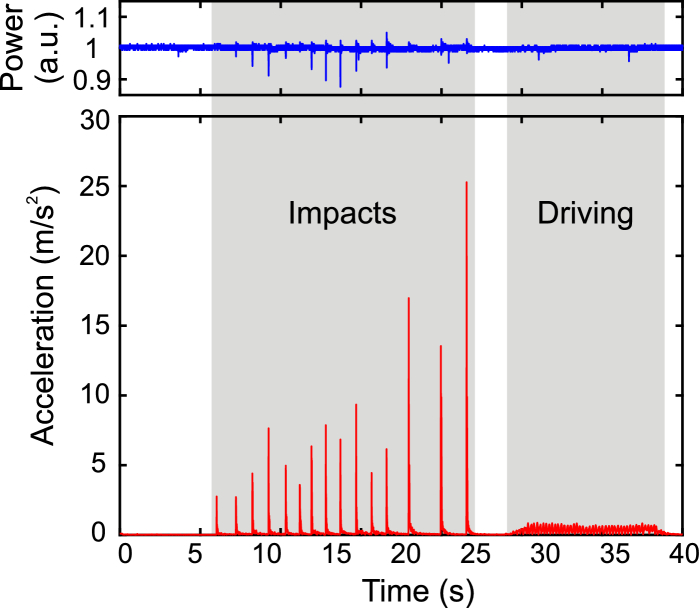
Results of the stress test of the light source placed on a laboratory cart. Lower panel shows the acceleration applied to the cart, grouped into impacts onto the cart and vibrations due to movements of the cart. Upper panel shows the simultaneously measured output signal power of the light source.

In order to support the mobile operation of CRS, we fitted the light source into a portable, passively cooled housing with a compact footprint of 50x40x15 ${\text{cm}}^{3}$. Electronics and pump diodes were cooled by a single processor fan in a separated standard 19 inch rack-mountable enclosure. In order to demonstrate the non-stationary applicability of this light source, we placed it on a simple laboratory cart and evaluated its operation during movements typical for transportation and handling. Figure 5

shows the acceleration applied to the cart, measured with a three-axis accelerometer, and the output power of the FOPO, simultaneously measured with a photodiode (low-pass filtered to a bandwidth of 500 kHz). Shocks with an acceleration of up to 25 $\text{m}/{\text{s}}^{2}$, which arose when inconsiderably handling the cart or bumping the cart into a wall at typical walking speed, did result in fluctuations of up to only 10 % of the output power. Right after the perturbation the FOPO inherently returned to its previous state, ensuring a reliable operation after careless handling. Arbitrarily moving the cart across our hallway resulted in vibrations of up to 1 $\text{m}/{\text{s}}^{2}$, which resulted in a noise level of the measured output power not higher than 1 %. The shock resistance and the turnkey operation of the laser system under real-world conditions were also successfully demonstrated by the laser system being immediately ready for measurements without the need for any readjustments or fine tuning after transporting the system over thousands of kilometer by car and airplane between the University of Münster (Germany) and the Wellmann Center for Photomedicine (Boston, USA). With this reliable performance and the compact footprint, the system can be readily integrated into a mobile cart, similar to intraoperative ultrasound or anesthesia carts. Due to the computer control of the laser source and the fiber delivery of the laser pulses to the microscope, no technical expertise is required for operation and the system constitutes an important step towards the establishment of CRS imaging techniques outside of laser laboratories.

## 3. Methods

In a first demonstration of the presented light source for biomedical relevant imaging, we applied the light source for CARS microscopy of dermal structures in human and animal skin. CARS microscopy is commonly used for dermatological investigations [32] including the evaluation of skin conditions [33,34], drug screening [35,36], and cancer diagnostics [8,37,38]. The presented imaging sessions were conducted with a preliminary version of the FOPO, which emitted pump and signal pulses with a duration of 7 and 3 ps, respectively, and a lower signal power of 70 mW.

### 3.1 Microscopy

The output signal pulses of the FOPO (tunable from 780 to 980 nm) were used as pump pulses and the amplified pulses from the pump laser (tunable between 1020 and 1060 nm) were used as Stokes pulses for CARS microscopy. Pump and Stokes pulses were overlapped in space and in time and both beams were conditioned in divergence with telescopes, and finally coupled into a commercially available inverted microscope (IX-81, Olympus, Tokyo, Japan), which was equipped with a confocal scanner (FV1000, Olympus, Tokyo, Japan). CARS microscopy was performed by relaying the overlapped pump and Stokes pulses to the back focal plane of a 20x 0.75 NA microscope objective (UPLSAPO 20X, Olympus, Tokyo, Japan). Laser powers were adjusted to yield 15 mW of average power for each the pump and the Stokes beams at the focus. The anti-Stokes signal was collected in the epi-direction, optically filtered with a shortpass dichroic mirror (750dcxr, Chroma, VT, USA) and a 60 nm bandpass filter centered at 650 nm (FF01-650/60-25, Semrock, NY, USA), and focused onto a photomultiplier tube (H7422PA-50, Hamamatsu Photonics, Hamamatsu City, Japan) that was biased at 0.7 kV and amplified with an RF amplifier (HCA-400k-50M, FEMTO, Berlin, Germany) for analog detection. Beam scanning and image acquisition were realized through a commercial Olympus Fluoview (FV10-ASW) software. Images of 512 x 512 pixels were acquired with 4 μs pixel dwell time with four Kalman averages per frame. Three-dimensional (3D) images were acquired by moving the objective lens in z-direction by 1 μm between consecutive frames. The images were analyzed with the open-source Fiji package [39]. A despeckle filter, which replaces each pixel with the median value in its 3 x 3 pixels’ neighborhood, was applied to all images for denoising. Multispectral images were false-colored and the ”Merge Channels” tool in Fiji was used to display different spectral components in a composite image. Volumes were projected into 2D images by color-coding of the consecutive frames with an Ice lookup table via the Temporal Color-code function.

### 3.2 Sample preparation

Human tissue samples were derived from deidentified exempt discarded facial skin tissue under MGH protocol 2017P002396. Obtained tissue blocks were embedded in an optimal cutting temperature medium and microtombed to yield 30 μm thin sections. Tissue was then placed on a microscope cover slide (pink slides) and imaged directly without a coverslip. Mouse tissue samples were obtained from freshly sacrificed and discarded Nu/Nu nude mice under MGH protocol 2017N000225. Mouse ears were gently clipped off and wiped with a lens paper soaked with PBS. Excised ears were then placed in a glass bottom dish (MatTek P35G-0-14-C) untreated or with 5 μL of dDMSO applied to the surface for immediate imaging. Colloidal imaging samples were created from commercial olive oil and deuterated dimethyl sulfoxide (DLM-10-10X0.75, Cambridge Isotope Laboratories Inc, MA, USA). Ruxolitinib was also commercially obtained and imaged in powder form directly on a microscope slide with coverslip.

## 4. Microscopy results and discussion

### 4.1 Ex vivo tissue tomography and comparison with a commercial system

In order to demonstrate chemical contrast and z-sectioning capability for medical applications *ex vivo* murine ear tissue acquired from nude mice, containing several dermal features similar to human skin, was imaged. Consecutive xy-planes were visualized with CARS tuned to 2845 ${\mathrm{cm}}^{-1}$ corresponding to the symmetric ${\mathrm{CH}}_{2}$-stretch vibrations of lipids. In order to evaluate the results obtained with the FOPO, the same regions of interests (ROI) were imaged following the same procedure (for details see section 3.) with a reference light source (Insight DS+, Spectra-Physics, CA, USA) including a femtosecond oscillator and a parametric frequency conversion stage. The dual-output reference source generated pulsed light at 80 MHz with approximately 100 to 200 fs pulse duration. One output of the reference source was fixed at 1041 nm and used as a Stokes beam, while the other was set to 803 nm to match the vibrational signature of the symmetric ${\mathrm{CH}}_{2}$-stretch at 2845 ${\text{cm}}^{-1}$. Although the excitation bandwidth of the femtosecond pulses (approx. 145 ${\text{cm}}^{-1}$, i.e., 8 nm) was too broad for resolving multiple Raman resonances in hyperspectral imaging, the femtosecond pulses were well suited to gain signal from the spectrally broad CH-stretch region. As the high peak power supplied by femtosecond light sources enables high nonlinear signal generation, femtosecond light sources, as represented by the reference source, are commonly used for label-free morphological imaging. This difference in functionality between the reference source and the presented narrowband picosecond system in respect of strong signal generation and high spectral resolution will inherently result in quantitatively different images. A comparison is, therefore, not quantitatively significant, but the images taken with the reference source from the same field of view act as a ground truth to show the expected morphological image content, i.e., sebaceous glands, adipocytes and subcutaneous fat.

Figure 6

**Fig. 6.**
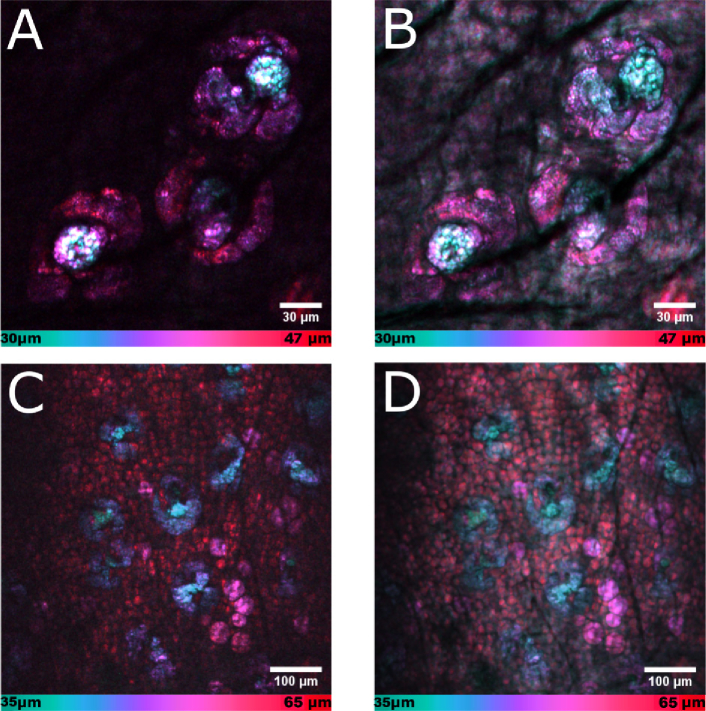
Color-depth projections of 3D volumes of fresh *ex vivo* mouse ear tissue imaged with lipid contrast (Ω = 2845 ${\mathrm{cm}}^{-1}$) CARS. Reference point of the depth color code bars at the bottom of each image is the skin surface at 0 μm. Volumes were consequently obtained with the fiber-based light source (a, c) and a reference light source (b, d). (a, b) Lipid-rich sebaceous glands. (c, d) Dermal structures from the sebaceous glands to adipocytes, and subcutaneous fat. The spectrally broadband excitation with the femtosecond reference source resulted in a higher overall brightness but lower image contrast due to the stronger non-resonant signal contribution in (b) and (d).

shows a region of interest about 30 μm below the skin surface, imaged with (a) the FOPO and (b) the reference light source. In both images sebaceous glands can be clearly distinguished, which consist of lipid-rich sebocytes packed with sebum granules. Boundaries between individual sebocytes along with (dark) nuclei are well defined. In the center of the glands (depicted in light blue) individual sebum granules secreted by matured sebocytes can be observed. The approximately two-fold higher signal-to-background ratio in the region of the lipid-rich sebaceous glands in Fig. 6 (a) is a result of the reduced non-resonant signal contribution due to the spectrally more narrow excitation bandwidth of the FOPO (approx. 12 ${\text{cm}}^{-1}$). Figure 6 furthermore shows color-coded tomograms of ear skin tissue acquired from approximately 30 to 60 μm below the surface with (c) the FOPO and (d) the reference source. As above, image content obtained with the portable FOPO is equivalent to the image content obtained with the stationary reference light source. Going from the surface down, sebaceous glands (blue) are followed by dermal adipocytes (magenta) and a layer of subcutaneous fat (red). The uniform haze, effectively reducing image contrast in Fig. 6 (d), was due to the stronger non-resonant background as a result of the broader excitation bandwidth of the reference source in comparison to the FOPO. Resolving these dermal structures (Fig. 6 (c)) as well as features of pilosebaceous units (Fig. 6 (a)) is a key aspect for dermatological investigations, e.g., of dynamic changes during the hollocrine secretion as affected by disease or therapy [40,41], and has verified that the fiber-based light source compares well with a state-of-the-art reference source.

### 4.2 Spectrally resolved coherent Raman imaging

**Fig. 7.**
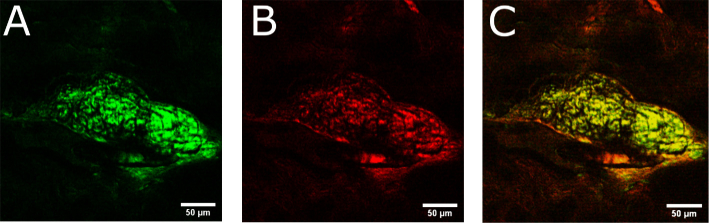
Images of human sebaceous gland from 30 μm thin skin tissue sections. (a) CARS image obtained by tuning to 2845 ${\mathrm{cm}}^{-1}$ (symmetric ${\mathrm{CH}}_{2}$). (b) CARS image obtained by tuning to 2934 ${\mathrm{cm}}^{-1}$ (asymmetric ${\mathrm{CH}}_{3}$). (c) Merged two-color image from (a) and (b) revealing heterogeneous distributions of lipids (green/yellow) and proteins (orange/red).

Besides its reliability and simplicity, a major appeal of using the fiber-based light source for CRS is its automated rapid and wide spectral tuning capability. In order to visualize rapidly evolving samples or take optical biopsies of living patients with high chemical specificity, video-rate hyperspectral imaging is required. However, the limited tuning speed of conventional laser systems (tuning time per step of more than 1 s) limits the frame rate in hyperspectral imaging to less than 1 Hz, leading to motion blurring and preventing image registration. A tuning time that is negligible compared to the frame acquisition time is required. For a first demonstration of the applicability of the FOPO towards hyperspectral imaging, we have measured the distribution of lipids and proteins in thin human skin tissue sections by switching the excitation wavelength in CARS between the ${\text{CH}}_{2}$- and ${\text{CH}}_{3}$-vibrations without the need for power and relative delay adjustments within only 5 ms. With this tuning speed, up to 100 user-selectable Raman resonances could be imaged per second, when imaging at speeds of 100 frames/s and assuming an equal time span for tuning and acquisition. For the here presented imaging session, human skin tissues were vertically sectioned into 30 μm slices spanning approximately 1 mm deep from the epidermis into the dermis. Fig. 7

shows the cross-section of a human sebaceous gland acquired from a 30 μm thick section. The pump and Stokes pulses derived from the FOPO allowed to separately image the individual distributions of ${\mathrm{CH}}_{2}$- (Fig. 7 (a)) and ${\mathrm{CH}}_{3}$-components (Fig. 7 (b)). The center of the gland is seen to be full of the lipid-rich sebocytes, while the periphery of the gland is constructed predominantly of pre-sebocytes, epithelial cells, and structural proteins such as collagen fibers. The acquisition of the shown multispectral data was straightforward due to the rapid all-electronic tuning offered by the FOPO without the need for moving parts or temperature changes.

**Fig. 8.**
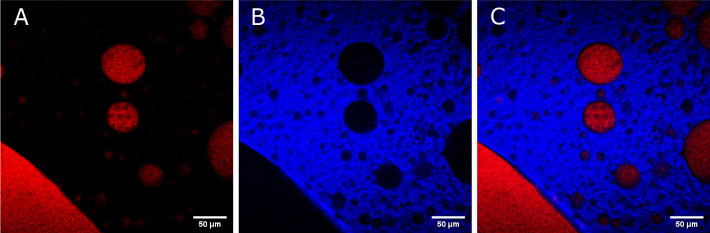
Images of a colloidal mixture of dDMSO and olive oil. (a) CARS image obtained by tuning to 2130 ${\mathrm{cm}}^{-1}$ (symmetric CD). (b) CARS image obtained by tuning to 2845 ${\mathrm{cm}}^{-1}$ (symmetric ${\mathrm{CH}}_{2}$). (c) Merged two-color image from (a) and (b) revealing discrete distributions of lipids and deuterated DMSO.

**Fig. 9.**
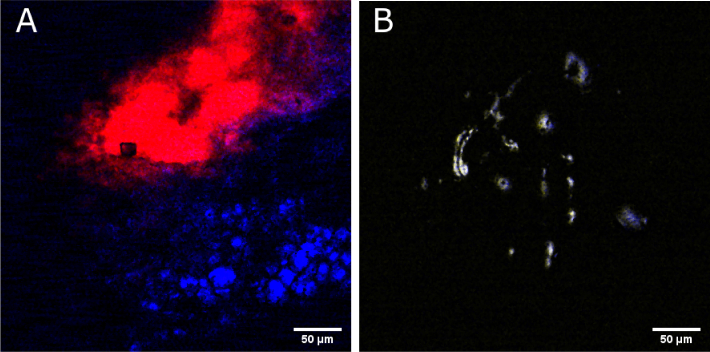
Images of selective wavelength tuning of FOPO between high wavenumber and silent region of Raman spectrum. (a) Merged two-color CARS image obtained by tuning to the 2130 ${\mathrm{cm}}^{-1}$ vibration (symmetric CD, red) of deuterated DMSO and the 2845 ${\mathrm{cm}}^{-1}$ vibration (symmetric ${\mathrm{CH}}_{2}$, blue) of subcutaneous fat following the application of dDMSO to nude mouse ear tissue. (b) Merged two-color CARS image of ruxolitinib powder obtained by tuning to 2250 ${\mathrm{cm}}^{-1}$ (CN stretching, yellow) and 2845 ${\mathrm{cm}}^{-1}$ (symmetric ${\mathrm{CH}}_{2}$, blue).

In order to demonstrate the imaging capability of the presented system outside of the CH-stretch region, we applied the system for imaging the distribution of lipids and deuterated dimethyl sulfoxide (dDMSO) both in solution and on mouse ear tissue with CARS microscopy. Rapid switching of the excitation wavelength to target 2130 ${\text{cm}}^{-1}$ (CD-vibration) and 2845 ${\text{cm}}^{-1}$ (${\mathrm{CH}}_{2}$-vibration) allowed, without the need for a relative delay adjustment, individual imaging of dDMSO droplets (red, Fig. 8 (a)

) in a colloidal mixture of lipids (blue, Fig. 8 (b)). Due to low pulse-to-pulse amplitude fluctuations of both the pump and Stokes pulses below 1 % RMS, a signal-to-noise ratio of more than 1600 in a homogenous ROI of dDMSO was achieved. A more relevant sample for biomedical imaging is presented in Fig. 9 (a) showing the distribution of dDMSO applied to *ex vivo* murine ear tissue. Here, the dDMSO (2130 ${\text{cm}}^{-1}$ CD-vibration) has penetrated from the surface of the skin and pooled within a fold within the papillary dermis (red) with adjacent subcutaneous fat (2845 ${\text{cm}}^{-1}$ ${\mathrm{CH}}_{2}$-vibration, blue) at a plane about 60 μm deep in the skin tissue. Fig. 9 (b)

depicts the colocalization of CN (2250 ${\text{cm}}^{-1}$, yellow) and CH (2845 ${\text{cm}}^{-1}$, blue) bonds within the crystalline structures of ruxolitinib, a commercial chemotherapy agent, demonstrating the selective tuning of this laser system to specific vibrations. Differentiating lipids and deuterated components with high specificity is a key requirement of several biomedical investigations, such as drug screening [20] or imaging of metabolic changes in organisms [22], and has not been shown with a mobile all-fiber system before.

## 5. Summary

In summary, we have presented an all-fiber light source for coherent Raman scattering microscopy, tunable across more than 2700 ${\text{cm}}^{-1}$ in less than 5 ms. Compared to previously presented fiber optical parametric oscillators, the system ran at a high repetition rate around 40 MHz for rapid imaging. The Stokes and pump pulses exhibited equal durations of 7 ps and an average power of up to 400 and 170 mW, respectively. The wide and rapid tunability was achieved by a novel tuning concept for OPOs, based on a rapidly tunable filter and a specifically chirped fiber Bragg grating as the output coupler, both within the master laser pumping the OPO. With the dispersive matching of master laser and parametric oscillator by the fiber Bragg grating, the repetition frequency change of the master laser equaled the corresponding repetition frequency change of the resonant signal pulses in the FOPO, such that both resonators stayed synchronized across the whole wavelength tuning range, without any alteration of the FOPO, e.g., via a mechanical delay line. Accordingly, the light source could be assembled out of all-spliced fiber components and the whole system fitted into a compact housing with a footprint of 50x40x15 ${\mathrm{cm}}^{3}$. The lack of free-space components and free-space propagation sections minimized disturbance by physical forces enabling operation even under mechanical acceleration of up to 25 $\text{m}/{\text{s}}^{2}$. Given the laser’s robust operation, the future applicability of the fiber-based light source in challenging environments like medical practices, clinics, and operating rooms can be expected. Additionally, the shown turn-key operation and the small footprint of the presented light source are key elements to build portable, cart-based systems for practical applications within the life sciences. This potential is further supported by the verification that the fiber-based light source was able to capture chemically selective and label-free CARS images with high spatial and spectral resolution with up to two orders of magnitude faster tunability compared to state-of-the-art reference light sources. In the future, we will characterize the noise performance of the light source in respect to its application within stimulated Raman scattering microscopy.

Altogether, the presented FOPO system represents an enabling technology for *in vivo* investigations in pharmaceutical and cosmetic research and for bed-side virtual optical biopsies. This source can become a crucial element for applying coherent Raman scattering imaging in clinical settings, while at the same time improving routine coherent Raman imaging in standard laboratory settings.
